# Enhancing Activity Engagement to Optimize Medicare Home Health Patient Outcomes: A Pilot RCT

**DOI:** 10.1093/geroni/igaf122.3925

**Published:** 2025-12-31

**Authors:** Chiung-ju Liu, Dorian Rose, Peihua Qiu, Zibo Tian, Alexandra Armstrong, Yvonne Lu

**Affiliations:** University of Florida College of Public Health and Health Professions, Gainesville, Florida, United States; University of Florida, Gainesville, Florida, United States; University of Florida, Gainesville, Florida, United States; University of Florida, Gainesville, Florida, United States; Duke University, Durham, North Carolina, United States; Indiana University School of Nursing, Indianapolis, Indiana, United States

## Abstract

Reducing task demands early in rehabilitation and gradually increasing them may enhance functional outcomes for patients receiving home health therapy. This pilot randomized controlled trial, completed in March 2025, examined the feasibility and preliminary effects of the Home-based Activity Reactivation Program, which integrates compensatory and restorative strategies to calibrate task demands as an adjuvant treatment to usual therapy. Forty-seven patients (mean age 82) were randomized to receive the Home-based Activity Reactivation Program plus usual therapy (n = 24) or usual therapy alone (n = 23). The Home-based Activity Reactivation Program was delivered in six weekly home visits, separate from usual therapy sessions. The primary outcome was Motor Skills from the Assessment of Motor and Process Skills; secondary outcomes included multiple measures of physical function. The Home-based Activity Reactivation Program demonstrated high acceptability, with 95% of completers reporting satisfaction and no severe or serious adverse events. Unexpectedly, the control group showed greater improvement in Motor Skills at postintervention (estimated difference -0.29 logits, p = 0.02), though this was not sustained at one-month follow-up. No significant between-group differences were found over time for secondary outcomes. These findings suggest that the Home-based Activity Reactivation Program did not yield superior outcomes compared to usual therapy alone. Potential contributing factors include limited intervention intensity, scheduling challenges, and practitioner bias. The study highlights the complexities of integrating and evaluating adjuvant interventions within routine home health care and underscores the need for further refinement and testing of the Home-based Activity Reactivation Program to optimize its effectiveness.

